# Supplementary material for: Ligand-specific activation trajectories dictate GPCR signalling in cells
Source: Nature. 2026 Jan 14;650(8103):1053–62. doi: 10.1038/s41586-025-09963-3 (PMC12935549; doi:10.1038/s41586-025-09963-3)
Supplement: Supplementary file 1 — Supplementary Figs. 1–6 and Supplementary Tables 1–7. The Supplementary Figures provide data on biosensor validation (labelling, order of ligand addition, G-protein overexpression). The Supplementary Tables list technical parameters (normalized fluorescence intensities, EC50, Emax, on-rates), primer sequences and G-protein subunits [file 41586_2025_9963_MOESM1_ESM.pdf]

nature portfolio

https://doi.org/10.1038/s41586-025-09963-3

Supplementary information

Ligand-specific activation trajectories dictate GPCR signalling in cells

In the format provided by the
authors and unedited

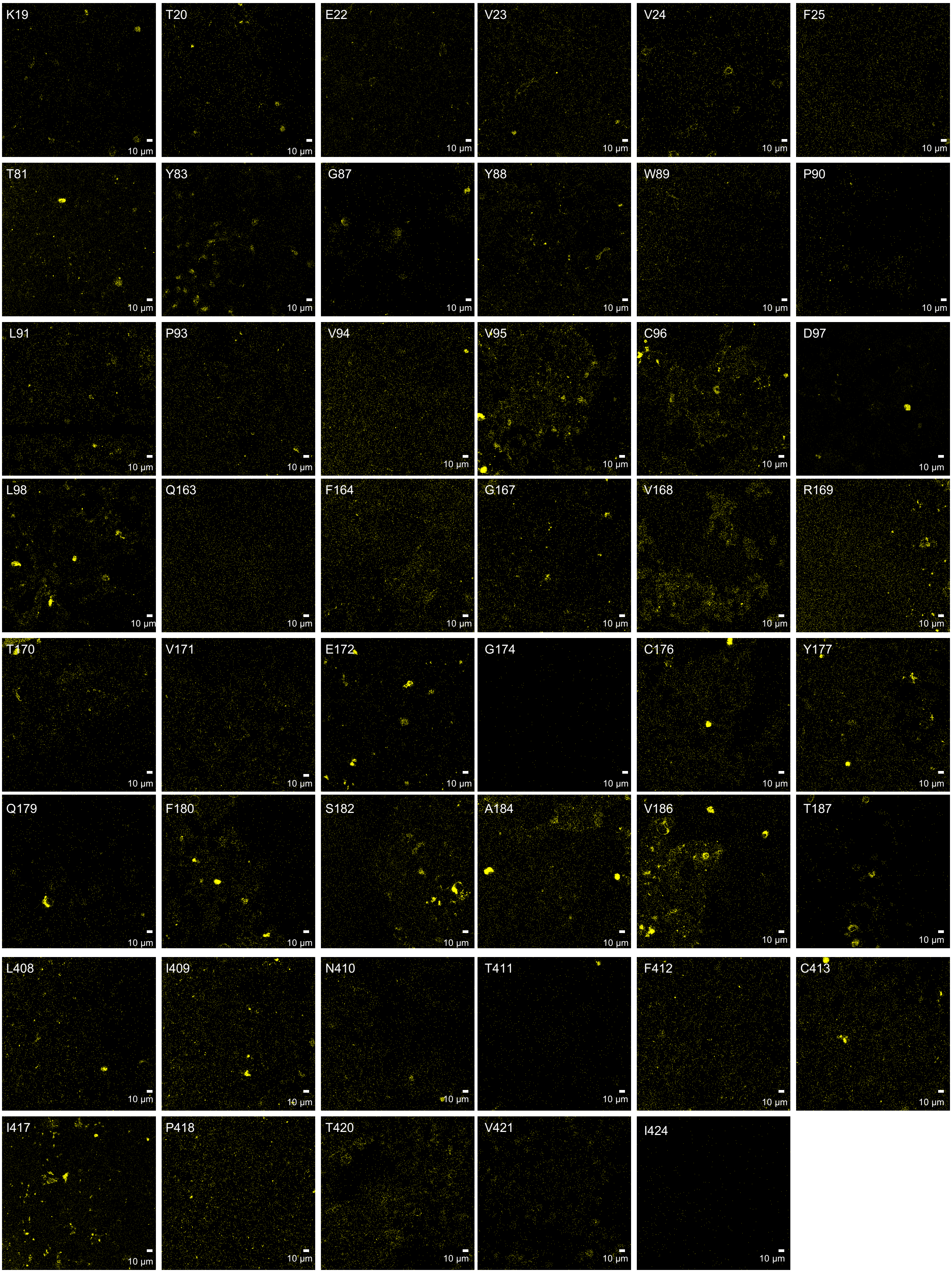
K19
10 µm
T20
10 µm
E22
10 µm
V23
10 µm
V24
10 µm
F25
10 µm
T81
10 µm
Y83
10 µm
G87
10 µm
Y88
10 µm
W89
10 µm
P90
10 µm
L91
10 µm
P93
10 µm
V94
10 µm
V95
10 µm
C96
10 µm
D97
10 µm
L98
10 µm
Q163
10 µm
F164
10 µm
G167
10 µm
V168
10 µm
R169
10 µm
T170
10 µm
V171
10 µm
E172
10 µm
G174
10 µm
C176
10 µm
Y177
10 µm
Q179
10 µm
F180
10 µm
S182
10 µm
A184
10 µm
V186
10 µm
T187
10 µm
L408
10 µm
I409
10 µm
N410
10 µm
T411
10 µm
F412
10 µm
C413
10 µm
I417
10 µm
P418
10 µm
T420
10 µm
V421
10 µm
I424
10 µm

Supplementary Figure 1: M_2R mutants that could not be labelled. Representative confocal images obtained from the labelling screen. The indicated M_2R mutants (SP-M_2R^{XXXTAG}) could not be labelled with Tet-Cy3 at the ncAA TCO*K incorporated at the indicated position (amino acid of the native human receptor). The data were obtained from at least 3 independent experiments.

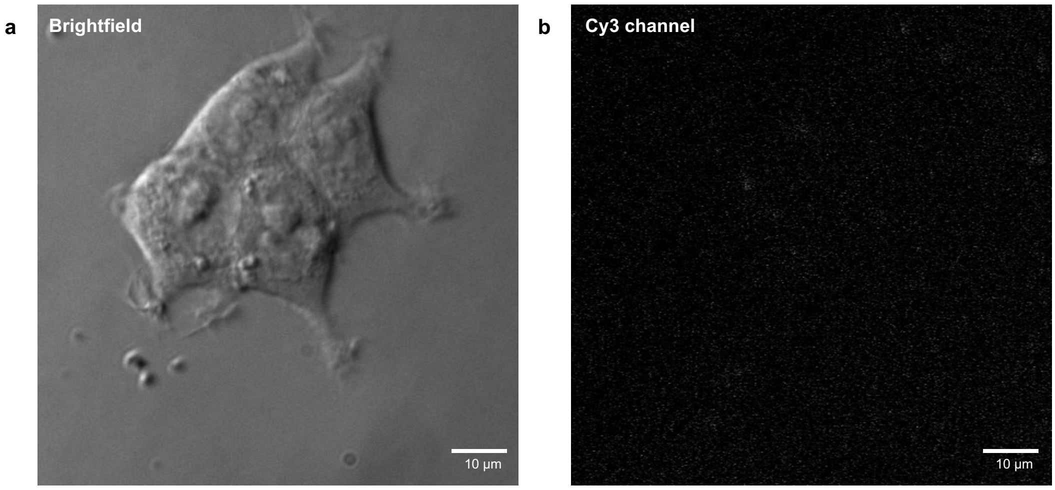

Supplementary Figure 2: Labelling of the M_2R^{414} biosensor in absence of the non-canonical amino acid TCO*K. Shown are representative epifluorescence images of HEK293T cells transfected with the cDNA of the tRNA synthetase, the tRNA cassette (*$MbPylRS^{AF}$/4x$tRNA^{M15}$*), and *SP-M_2R^{414TAG}* in absence of the ncAA TCO*K after labelling with Tet-Cy3. (a) brightfield, (b) Cy3 channel.

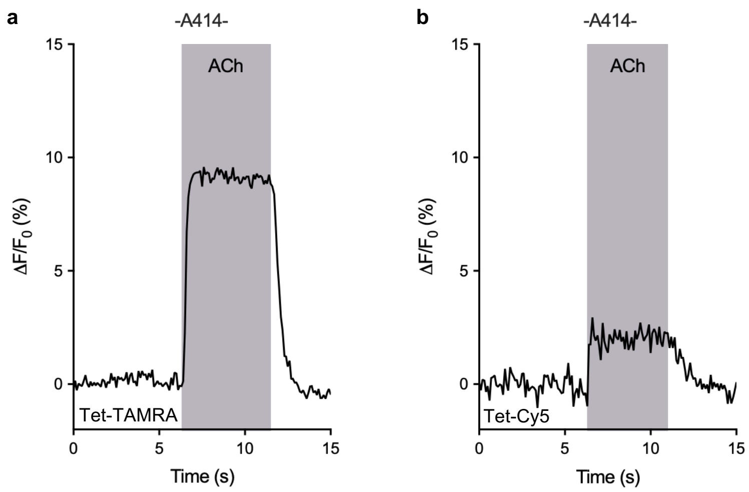

Supplementary Figure 3: Conformational changes can be monitored with fluorescent dyes of different chemistry. Representative traces of $\Delta F/F_0$ of single cells expressing the M_2R-A414 biosensor (*SP-M₂R*^{A414TAG}) and labelled with Tetrazine-TAMRA (a) or Tetrazine-Cyanine5 (b) stimulated with 1 mM ACh. Shaded areas indicate the duration of agonist superfusion, unshaded areas represent buffer application. Data obtained from 3 independent experiments. Number of cells (n): n = 31 (a), n = 27 (b).

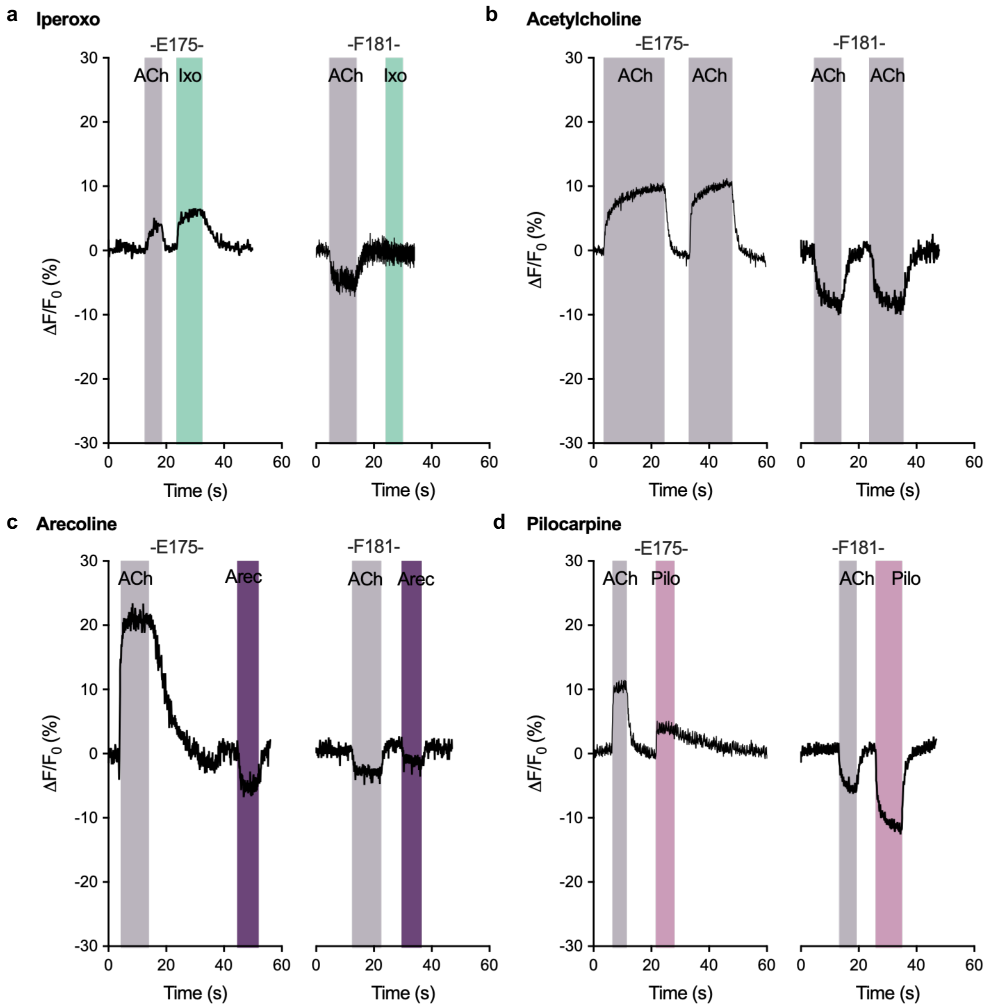

Supplementary Figure 4: Agonist efficacies at biosensors are independent of the order of ligand addition. Representative fluorescence intensity changes ($\Delta F/F_0$) recorded in real-time from single HEK293T cells expressing indicated M_2R biosensors (*SP-M₂R*^{XXXTAG}) superfused with 1mM ACh, followed by (a) 100 µM iperoxo (Ixo), (b) 1mM ACh, (c) 1 mM arecoline (Arec), or (d) 10 mM pilocarpine (Pilo), after washout with buffer, respectively. Application of different agonists is indicated with shaded areas in different colors. Non-shaded areas indicate buffer application. Number of individual cells (n, for Ixo, ACh, Arec, Pilo): E175 (n = 29, 54, 15, 22); F181 (n = 20, 68, 22, 14). Number of independent experiments (for Ixo, ACh, Arec, Pilo): E175 = 5, 9, 3, 3; F181 = 3, 12, 3, 3.

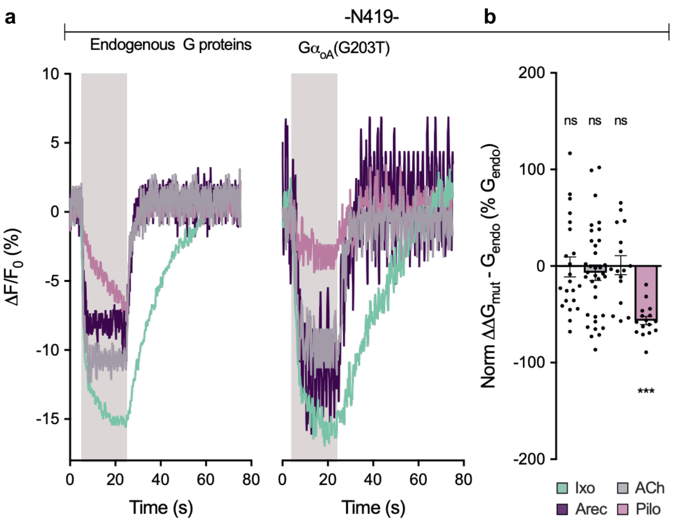

Supplementary Figure 5: Conformational changes at M_2R-N419 are independent of G-protein activity. (a) Representative traces of $\Delta F/F_0$ of single cells expressing the M_2R-N419 biosensor (*SP-$M_2R^{N419TAG}$*) at endogenous G-protein levels (left) or after $G\alpha_{oA}$(G203T) overexpression (center) and stimulated with 1 mM ACh (grey), 100 µM Ixo (green), 1 mM Arec (purple), or 10 mM Pilo (pink). Shaded areas indicate the duration of agonist superfusion, unshaded areas represent buffer application. (b) Statistical summary of the normalised changes in $\Delta F/F_0$ obtained from experiments in (a). Shown are the ligand-dependent differences in $\Delta F/F_0$ after $G\alpha_{oA}$(G203T) overexpression (G_{mut}) normalised to endogenous G-protein levels (G_{endo}, set to 0%). Negative values indicate decreases in $\Delta F/F_0$ after $G\alpha_{oA}$(G203T) overexpression, and positive values indicate increases in $\Delta F/F_0$ after $G\alpha_{oA}$(G203T) overexpression. The bars represent means ± sem, each data point represents a single cell. n, number of cells, number of independent experiments: n = 13, 3 (Ixo), n = 24, 4 (ACh), n = 13, 4 (Arec), n = 14, 3 (Pilo). ***$p < 0.001$ (0.0004), according to an unpaired two-tailed t-test. G_{endo}, endogenous G proteins; mut, mutant; ns, not significantly different.

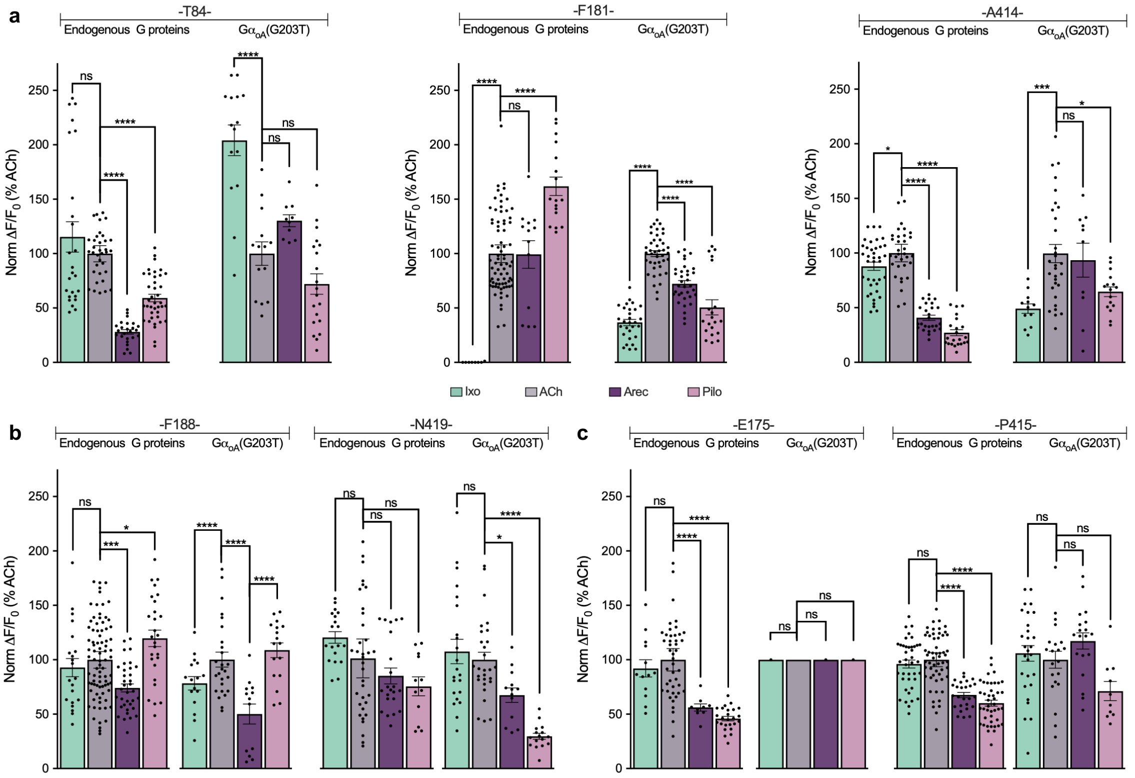

Supplementary Figure 6: $G\alpha_{oA}$(G203T) overexpression changes the equilibrium of receptor/G-protein complexes. Comparison of the ligand-dependent differences in $\Delta F/F_0$ after $G\alpha_{oA}$(G203T) overexpression or at endogenous G-protein levels after activation of the indicated M_2R biosensors (*SP-M_2R^{XXXTAG}*) with the ligands 100 µM Ixo (green), 1 mM ACh (grey), 1 mM Arec (purple), and 10 mM Pilo (pink). For each condition, the data has been normalised to the mean $\Delta F/F_0$ of ACh (set to 100%). The bars represent means ± sem of at least 3 independent experiments, each data point represents a single cell. Number of individual cells (n, for Ixo, ACh, Arec, Pilo): n = 15, 14, 10, 20 (T84), n = 11, 10, 9, 11 (E175), n = 29, 41, 34, 20 (F181), n = 16, 26, 13, 17 (F188), n = 12, 31, 10, 16 (A414), n = 28, 21, 19, 10 (P415), n = 13, 24, 13, 14 (N419). ****$p < 0.0001$, ***$p < 0.001$, **$p < 0.01$, *$p < 0.05$ according to an one-way analysis of variance (ANOVA multiple comparisons test) with Tukey's posthoc test. ns, not significantly different.

Supplementary Table 1 (related to Figs. 1 and 2): Mean ΔF/F_0 [%] from M_2R biosensors stimulated with distinct agonists. N: total number of cells; Exp.: independent days of transfections; SEM: standard error of the mean.

	T84^{TM2}				E175^{ECL2}				F181^{ECL2}			
Agonist	Mean	SEM	N	Exp.	Mean	SEM	N	Exp.	Mean	SEM	N	Exp.
Ixo	9.36	1.20	21	7	9.61	0.83	13	7	0.00	0.01	12	5
ACh	8.46	0.31	35	17	10.43	0.54	44	11	-5.86	0.24	76	9
Arec	-2.38	0.18	25	6	-5.86	0.35	9	4	-5.82	0.75	13	7
Pilo	5.02	0.29	39	7	4.80	0.22	25	5	-9.50	0.50	17	7

	F188^{TM5}				A414^{ECL3}				P415^{ECL3}			
Agonist	Mean	SEM	N	Exp.	Mean	SEM	N	Exp.	Mean	SEM	N	Exp.
Ixo	-8.42	0.75	21	5	-14.4	0.61	38	12	-15.52	0.58	40	9
ACh	-11.10	0.52	96	15	-16.36	0.64	33	7	-16.15	0.52	56	9
Arec	-6.72	0.34	35	6	-6.68	0.38	26	11	-10.92	0.39	26	5
Pilo	-10.86	0.68	27	6	-4.44	0.46	22	3	-9.71	0.46	43	10

	N419^{TM7}			
Agonist	Mean	SEM	N	Exp.
Ixo	-13.76	0.61	19	9
ACh	-11.66	1.02	34	12
Arec	-9.72	0.83	22	6
Pilo	-8.63	0.99	12	6

Supplementary Table 2 (related to Fig. 2 and Extended Data Fig. 5: Potency (pEC_{50}) and maximum efficacy (E_{max}) determined by G_{oA} TRUPATH assays of wildtype M_2R or M_2R biosensors stimulated with the indicated agonists. Each condition was performed as 3-4 independent experiments. E_{max} indicates the BRET response normalised to the maximum BRET response of ACh (% of ACh) for the respective conditions of receptor and ligand. SEM: standard error of the mean.

	Wildtype M_2R							
Agonist	Ixo		ACh		Arec		Pilo	
	pEC_{50}	E_{max}	pEC_{50}	E_{max}	pEC_{50}	E_{max}	pEC_{50}	E_{max}
Mean	10.36	100.70	7.93	100.40	6.66	116.10	2.10	85.49
SEM	0.06	0.58	0.11	0.61	0.12	3.64	0.18	0.86

	$T84^{TM2}$							
Agonist	Ixo		ACh		Arec		Pilo	
	pEC_{50}	E_{max}	pEC_{50}	E_{max}	pEC_{50}	E_{max}	pEC_{50}	E_{max}
Mean	10.21	106.30	8.11	102.00	6.67	114.00	6.61	71.00
SEM	0.15	1.09	0.15	0.47	0.12	1.28	0.19	1.48

	$E175^{ECL2}$							
Agonist	Ixo		ACh		Arec		Pilo	
	pEC_{50}	E_{max}	pEC_{50}	E_{max}	pEC_{50}	E_{max}	pEC_{50}	E_{max}
Mean	10.32	108.40	8.25	101.3	7.02	120.2	6.28	82.48
SEM	0.17	0.90	0.14	1.54	0.14	1.49	0.22	2.03

	$F181^{ECL2}$							
Agonist	Ixo		ACh		Arec		Pilo	
	pEC_{50}	E_{max}	pEC_{50}	E_{max}	pEC_{50}	E_{max}	pEC_{50}	E_{max}
Mean	8.36	102.40	5.81	101.60	5.10	49.97	4.97	27.92
SEM	0.10	0.74	0.08	0.70	0.12	1.09	0.01	0.58

	$F188^{TM5}$							
Agonist	Ixo		ACh		Arec		Pilo	
	pEC_{50}	E_{max}	pEC_{50}	E_{max}	pEC_{50}	E_{max}	pEC_{50}	E_{max}
Mean	9.58	105.80	7.02	103.80	6.06	106.00	5.60	61.62
SEM	0.03	1.47	0.02	1.63	0.02	2.00	0.08	1.82

	$A414^{ECL3}$							
Agonist	Ixo		ACh		Arec		Pilo	
	pEC_{50}	E_{max}	pEC_{50}	E_{max}	pEC_{50}	E_{max}	pEC_{50}	E_{max}
Mean	10.81	106.00	8.60	99.90	7.90	113.80	6.57	83.52
SEM	0.09	1.14	0.06	1.16	0.09	1.25	0.05	1.93

	$P415^{ECL3}$							
Agonist	Ixo		ACh		Arec		Pilo	
	pEC_{50}	E_{max}	pEC_{50}	E_{max}	pEC_{50}	E_{max}	pEC_{50}	E_{max}
Mean	10.47	105.40	8.24	101.30	7.39	115.00	6.75	82.43
SEM	0.04	1.05	0.05	1.06	0.25	1.73	0.28	1.33

	$N419^{TM7}$							
Agonist	Ixo		ACh		Arec		Pilo	
	pEC_{50}	E_{max}	pEC_{50}	E_{max}	pEC_{50}	E_{max}	pEC_{50}	E_{max}
Mean	9.40	107.70	7.27	102.10	6.25	92.16	6.10	47.79
SEM	0.15	1.26	0.10	0.94	0.04	1.33	0.09	1.51

Supplementary Table 3 (related to Fig. 4 and Extended Data Fig. 9): Mean Tau-ON (apparent on-rates, s) values of fluorescence intensity changes from M_2R biosensors stimulated with the indicated agonists. N: total number of cells. Exp: days of independent transfections, SEM: standard error of mean, G_{endo}: endogenous G-protein level, $G\alpha_{oA}$: overexpressed $G\alpha_{oA}$(G203T) mutant.

	$T84^{TM2}$								$E175^{ECL2}$							
Agonist	Ixo		ACh		Arec		Pilo		Ixo		ACh		Arec		Pilo	
	G_{endo}	$G\alpha_{oA}$	G_{endo}	$G\alpha_{oA}$	G_{endo}	$G\alpha_{oA}$	G_{endo}	$G\alpha_{oA}$	G_{endo}	$G\alpha_{oA}$	G_{endo}	$G\alpha_{oA}$	G_{endo}	$G\alpha_{oA}$	G_{endo}	$G\alpha_{oA}$
Mean	1.12	1.06	0.25	0.64	0.34	2.72	2.10	1.14	1.03	NA	0.50	NA	0.27	NA	0.18	NA
SEM	0.11	0.09	0.02	0.15	0.06	0.60	0.18	0.16	0.14	NA	0.02	NA	0.03	NA	0.01	NA
N	25	15	35	14	25	10	39	20	13	11	44	10	9	9	25	11
Exp	7	5	17	7	6	3	7	4	7	3	11	3	4	3	5	3

	$F181^{ECL2}$								$F188^{TM5}$							
Agonist	Ixo		ACh		Arec		Pilo		Ixo		ACh		Arec		Pilo	
	G_{endo}	$G\alpha_{oA}$	G_{endo}	$G\alpha_{oA}$	G_{endo}	$G\alpha_{oA}$	G_{endo}	$G\alpha_{oA}$	G_{endo}	$G\alpha_{oA}$	G_{endo}	$G\alpha_{oA}$	G_{endo}	$G\alpha_{oA}$	G_{endo}	$G\alpha_{oA}$
Mean	NA	18.41	1.33	1.82	1.85	1.96	1.87	1.50	3.21	9.35	2.69	3.20	2.21	4.64	4.73	8.09
SEM	NA	1.48	0.04	0.04	0.16	0.13	0.10	0.32	0.26	0.45	0.11	0.12	0.14	0.45	0.34	0.54
N	12	29	76	41	13	34	17	20	21	16	96	26	35	13	27	17
Exp	5	4	9	7	7	4	7	3	5	3	15	4	6	3	6	3

	$A414^{ECL3}$								$P415^{ECL3}$							
Agonist	Ixo		ACh		Arec		Pilo		Ixo		ACh		Arec		Pilo	
	G_{endo}	$G\alpha_{oA}$	G_{endo}	$G\alpha_{oA}$	G_{endo}	$G\alpha_{oA}$	G_{endo}	$G\alpha_{oA}$	G_{endo}	$G\alpha_{oA}$	G_{endo}	$G\alpha_{oA}$	G_{endo}	$G\alpha_{oA}$	G_{endo}	$G\alpha_{oA}$
Mean	0.53	2.21	0.17	0.49	0.87	0.54	0.33	0.68	0.34	0.44	0.16	0.59	0.22	0.68	0.21	0.28
SEM	0.03	0.34	0.01	0.08	0.12	0.08	0.02	0.10	0.03	0.03	0.00	0.08	0.01	0.17	0.01	0.06
N	38	12	33	31	26	10	22	16	40	28	56	21	26	19	43	10
Exp	12	4	7	9	11	3	3	3	9	5	9	6	5	5	10	3

Supplementary Table 4: Mean pEC_{50} values of the concentration-effect-curves shown in Extended Data Fig. 10. N: total number of experiments; SEM: standard error of the mean.

	Acetylcholine			Iperoxo			Arecoline			Pilocarpine		
Gα protein	Mean	SEM	N	Mean	SEM	N	Mean	SEM	N	Mean	SEM	N
G_{i1}	7.27	0.15	4	9.30	0.10	6	6.00	0.11	4	5.18	0.13	6
G_{i2}	7.41	0.14	4	9.75	0.14	7	6.18	0.07	4	5.63	0.10	6
G_{i3}	6.55	0.16	4	8.74	0.10	6	5.68	0.10	4	5.36	0.22	5
G_{oA}	7.39	0.10	6	9.65	0.05	5	6.54	0.07	3	5.50	0.06	6
G_{oB}	7.76	0.12	5	9.83	0.10	8	6.66	0.12	3	5.82	0.10	5
G_z	6.56	0.15	5	8.90	0.15	6	5.85	0.09	4	4.77	0.28	4
G_{15}	6.77	0.23	6	9.50	0.10	6	5.68	0.21	4	5.44	0.29	5

Supplementary Table 5 (related to Methods): Primer sequences for Gibson assembly. Primer sequences are depicted as 5'-3' reading frame. The priming region is highlighted in capital letters.

#	Sequence (5'-3')
1	ATTCTAGTTGTGGTTTGTCCAAACTCATCAA
2	ggagtttgttgagttattGCTGCCGGCGAACACCAGGCAGAAGATGTAGCTCAGGGCGATGATCGTCTTCATCAGTGTGATGGATATCTG
3	TTGATGAGTTTGGACAAACCACAACTAGAAT
4	ataggcgctacaaggTAAGCGGCCGCTCGAGCAT
5	CAGATATCCATCACACTGATGAAGACGATCATCGCCCTGAGCTACATCTTCTGCCTGGTGTTCGCCGGCAGCaataactcaacaaactcc
6	ATGCTCGAGCGGCCGCTTAccttgtagcgcctat
7	ctcctcgcccttgctcacgcctccgccaccGGTCCTTGTAGCGCCTAT
8	atggacgagctgtacaagtaaTGAGCGGCCGCTCGA
9	aggaccggtggcggaggcGTGAGCAAGGGCGAGGAG
10	tcgagcggccgctcattaCTTGTACAGCTCGTCCAT
11	TTCTGCCTGGTGTTCGCCTACCCATACGATGTTCCAGATTACGCTGGCAGCaataactcaaca
12	tgttgagttattGCTGCCAGCGTAATCTGGAACATCGTATGGGTAGGCGAACACCAGGCAGAA
13	GACAATTGGTgctTGGCTTTGTTACATC
14	CACACAGTGTTGGGG

Supplementary Table 6: Primers for site-directed mutagenesis (related to Methods). The respective M_2R residue was exchanged by an amber stop codon (TAG) using the listed forward and reverse primers. Primer sequences are depicted in the 5'-3' reading frame. The TAG is highlighted in capital letters.

M_2R residue	Forward (5'-3')	Reverse (5'-3')
K19	TAGacatttgaagtggtgtttattgtcctggtg	cttcaaatgtCTAataaggacttgtaagagccag
T20	gTAGtttgaagtggtgtttattgtcctggtgg	cacttcaaaCTActtataaggacttgtaagagccag
F21	gacaTAGgaagtggtgtttattgtcctggtg	ccacttcCTAtgtcttataaggacttgtaagagc
E22	TAGgtggtgtttattgtcctggtggctgg	taaacaccacCTAaaatgtcttataaggacttgtaagag
V23	gaaTAGgtgtttattgtcctggtggctgg	taaacacCTAttcaaatgtcttataaggacttgtaagag
V24	gTAGtttattgtcctggtggctggatcc	ggacaataaaCTAcacttcaaatgtcttataaggacttg
F25	gtggtgTAGattgtcctggtggctgg	gacaatCTAcaccacttcaaatgtcttataaggacttg
T81	tgtacTAGctctacactgtgattggttactgg	gtagagCTAgtacaagttcatggagaaaacacc
L82	tgtacaccTAGtacactgtgattggttactgg	gtaCTAggtgtacaagttcatggagaaaacacc
Y93	gtacaccctcTAGactgtgattggttactg	CTAgagggtgtacaagttcatggagaaaacac
T84	cTAGgtgattggttactggcctttgggac	aaccaatcacCTAgtagagggtgtacaagttc
V85	ctTAGattggttactggcctttgggacc	gtaaccaatCTAagtgtagagggtgtacaag
I86	gtgTAGggttactggcctttgggacc	ccagtaaccCTAcacagtgtagagggtg
G87	TAGtactggcctttgggacctgtggtg	aaggccagtaCTAaatcacagtgtagagggtg
Y88	ggtTAGtggcctttgggacctgtgg	aaaggccaCTAaccaatcacagtgtagagg
W89	cTAGcctttgggacctgtggtgtg	gtcccaaaggCTAgtaaccaatcacagtg
P90	ctggTAGttgggacctgtggtgtgtg	ggtcccaaCTAccagtaaccaatcacagtg
L91	ggcctTAGggacctgtggtgtg	ggtccCTAaggccagtaaccaatcac
G92	ggcctttgTAGcctgtggtgtgtgac	ggCTAcaaaggccagtaaccaatcacagtg
P93	ggaTAGgtggtgtgtgacctttggctag	acaccacCTAtcccaaaggccagtaacc
V94	gggacctTAGgtgtgtgacctttgg	cacacCTAaggtcccaaaggccag
V95	gTAGtgtgacctttggctagccctg	aaggtcacaCTAcacaggtcccaaagg
C96	ggtgTAGgacctttggctagccctg	aaggtcCTAcaccacaggtcccaaagg
D97	ggtgtgtTAGctttggctagccctgg	caaagCTAacacaccacaggtcccaaagg
L98	gtgtgacTAGtggctagccctggac	gccaCTAgtcacacaccacaggtcc
F161	ctcTAGtggcagttcattgtaggggtgag	gaactgccaCTAgagaatggctggagc
W162	ctcttcTAGcagttcattgtaggggtgag	gaactgCTAgaagagaatggctggagc
Q163	ctcttctggTAGttcattgtaggggtgag	gaaCTAccagaagagaatggctggagc
F164	ctcttctggcagTAGattgtaggggtgag	Actgccagaagagaatggctggagc
I165	cTAGgtaggggtgagaactgtggagg	cacccctacCTAgaactgccagaagag
V166	TAGggggtgagaactgtggaggatgg	gttctcaccccCTAaatgaactgccagaag
G167	TAGgtgagaactgtggaggatggggag	cacagttctcacCTAtacaatgaactgccag
V168	gTAGagaactgtggaggatggggag	ccacagttctCTAccctacaatgaactgc
R169	ggtgTAGactgtggaggatggggag	ccacagtCTAcaccccctacaatgaactgc
T170	ggtgagaTAGgtggaggatggggag	ccacCTAtctcacccctacaatgaactgc
V171	gagaactTAGgaggatggggagtgc	cctcCTAagttctcacccctacaatgaac
E172	gaactgtgTAGgatggggagtgctac	catcCTAcacagttctcacccctacaatg
D173	ctgtggagTAGggggagtgctacattc	ccCTActccacagttctcacccctac
G174	ctgtggaggatTAGgagtgctacattcag	CTAatcctccacagttctcacccctac
E175	ggatgggTAGtgctacattcagtttttttcc	gcaCTAcccatcctccacagttctc
C176	ggaggatggggagTAGtacattcagtttttttc	ctccccatcctccacagttctcac
Y177	ggatggggagtgcTAGattcagtttttttcc	gcactccccatcctccacagttc
I178	tggggagtgctacTAGcagtttttttccaatg	gtagcactccccatcctccacag

Supplementary Table 6: Site-directed mutagenesis primers. - Continues

# M_2R residue	Forward (5‘-3‘)	Reverse (5‘-3‘)
Q179	ggggagtgctacattTAGttttttccaatgc	tgtagcactccccatcctccacag
F180	cattcagTAGttttccaatgctgctgtcacc	aaaCTActgaatgtagcactccccatcctcc
F181	TAGtccaatgctgctgtcacctttggtacg	cagcattggaCTAaaactgaatgtagcactcc
S182	gttttttTAGaatgctgctgtcacctttggtacg	cattCTAaaaaaactgaatgtagcactccccatcc
N183	ccTAGgctgctgtcacctttggtacg	gacagcagcCTAggaaaaaaactgaatgtagc
A184	TAGgctgtcacctttggtacggctattgc	aggtgacagcCTAattggaaaaaaactgaatgtagc
A185	gctTAGgtcacctttggtacggctattgc	aggtgacCTAagcattggaaaaaaactgaatgtagc
V186	gctgctTAGacctttggtacggctattgc	aggtCTAagcagcattggaaaaaaactgaatgtagc
T187	gctgctgtcTAGtttggtacggctattgc	aCTAgacagcagcattggaaaaaaactgaatgtagc
F188	gctgtcaccTAGggtacggctattgc	cCTAggtgacagcagcattggaaaaaaactg
G189	gctgtcacctttTAGacggctattgcagc	Aaaaggtgacagcagcattggaaaaaaactgaatg
V407	gtcatgTAGctcattaacaccttttgtgcac	tgagCTAcatgacattgtatggggcccaag
L498	gtcatggtgTAGattaacaccttttgtgcacc	tCTAcaccatgacattgtatggggcccaag
I409	catggtgctcTAGaacaccttttgtgcacc	CTAgagcaccatgacattgtatggggcccaag
N410	TAGaccttttgtgcaccttgcatccccaac	gcacaaaaggtCTAaatgagcaccatgacattg
T411	cTAGttttgtgcaccttgcatccccaacac	ggtgcacaaaaCTAgttaatgagcaccatgac
F412	ccTAGtgtgcaccttgcatccccaac	ggtgcacaCTAggtgttaatgagcaccatg
C413	TAGgcaccttgcatccccaacactgtg	tgcaaggtgcCTAaaaggtgttaatgagcac
A414	TAGccttgcatccccaacactgtgtgg	ggatgcaaggCTAacaaaaggtgttaatgagc
P415	gcaTAGtgcatccccaacactgtgtgg	ggatgcaCTAtgcacaaaaggtgttaatgagc
C416	gcacctTAGatccccaacactgtgtgg	ggggatCTAaggtgcacaaaaggtgttaatg
I417	gcaccttgcTAGcccaacactgtgtg	gggCTAgcaaggtgcacaaaaggtgttaatg
P418	gcaccttgcatcTAGaacactgtgtggac	Agatgcaaggtgcacaaaaggtgttaatgag
N419	gcatccccTAGactgtgtggacaattgg	gtCTAggggatgcaaggtgcacaaaagg
T420	gcatccccaacTAGgtgtggacaattgg	TAgttggggatgcaaggtgcacaaaagg
V421	ccccaacactTAGtggacaattggttactg	CTAagtgttggggatgcaaggtgcac
W422	ccccaacactgtgTAGacaattggttactg	cacagtgttggggatgcaaggtgc
T423	aacactgtgtggTAGattggttactggctttg	Accacacagtgttggggatgcaagg
I424	cactgtgtggacaTAGggttactggctttg	tgtccacacagtgttggggatgcaag

Supplementary Table 7: G-protein subunits for TRUPATH G-protein activation assays. (related to Methods). Gβ and Gγ subunits used for the respective Gα subunit. The combination of subunits has been previously optimized for each Gα by Olsen et al[54].

Gα	Gγ	Gβ
i1	γ9	β3
i2	γ8	β3
i3	γ9	β3
oA	γ8	β3
oB	γ8	β3
Z	γ1	β3
Gust	γ1	β3
sS	γ9	β3
sL	γ1	β1
11	γ13	β3
12	γ9	β3
13	γ9	β3
15	γ13	β3
