# Supplementary material for: Ligand-specific activation trajectories dictate GPCR signalling in cells
Source: Nature. 2026 Jan 14;650(8103):1053–62. doi: 10.1038/s41586-025-09963-3 (PMC12935549; doi:10.1038/s41586-025-09963-3)
Supplement: Supplementary file 2 — Reporting Summary [file 41586_2025_9963_MOESM2_ESM.pdf]

nature portfolio

Corresponding author(s): Irene Coin
Andreas Bock

Last updated by author(s): Nov 19, 2025

Reporting Summary

Nature Portfolio wishes to improve the reproducibility of the work that we publish. This form provides structure for consistency and transparency in reporting. For further information on Nature Portfolio policies, see our Editorial Policies and the Editorial Policy Checklist.

Statistics

For all statistical analyses, confirm that the following items are present in the figure legend, table legend, main text, or Methods section.

n/a	Confirmed	
☐	☒	The exact sample size (*n*) for each experimental group/condition, given as a discrete number and unit of measurement
☐	☒	A statement on whether measurements were taken from distinct samples or whether the same sample was measured repeatedly
☐	☒	The statistical test(s) used AND whether they are one- or two-sided<br>*Only common tests should be described solely by name; describe more complex techniques in the Methods section.*
☒	☐	A description of all covariates tested
☐	☒	A description of any assumptions or corrections, such as tests of normality and adjustment for multiple comparisons
☐	☒	A full description of the statistical parameters including central tendency (e.g. means) or other basic estimates (e.g. regression coefficient) AND variation (e.g. standard deviation) or associated estimates of uncertainty (e.g. confidence intervals)
☐	☒	For null hypothesis testing, the test statistic (e.g. *F*, *t*, *r*) with confidence intervals, effect sizes, degrees of freedom and *P* value noted<br>*Give P values as exact values whenever suitable.*
☒	☐	For Bayesian analysis, information on the choice of priors and Markov chain Monte Carlo settings
☒	☐	For hierarchical and complex designs, identification of the appropriate level for tests and full reporting of outcomes
☒	☐	Estimates of effect sizes (e.g. Cohen's *d*, Pearson's *r*), indicating how they were calculated

Our web collection on statistics for biologists contains articles on many of the points above.

Software and code

Policy information about availability of computer code

Data collection	Single-cell fluorescence microscopy data were collected with VisiView 4.0 software (Visitron Systems), temporal brightness data with LASX software 3.5.7.23225 (Leica Biosystems), Images from internalisation experiments were collected with the Zen blue 2.3 lite software (Zeiss), the Gi3-FRET activation with MicroManager 2.0, TRUPATH data with Gen5 3.11 software (Agilent Technologies), and ELISA data were collected with EnVision Manager 1.13.3.
Data analysis	The following published software was used to analyse the data as referenced in Methods: Origin2022b 9.9.9.171 (OriginLab); ImageJ 1.5.4f, (NIH), GraphPad Prism 7.0 or newer (Graphpad Software), Microsoft Excel 2016, Python 3.7.9 (Python software foundation).

For manuscripts utilizing custom algorithms or software that are central to the research but not yet described in published literature, software must be made available to editors and reviewers. We strongly encourage code deposition in a community repository (e.g. GitHub). See the Nature Portfolio guidelines for submitting code & software for further information.

Data

Policy information about availability of data

All manuscripts must include a data availability statement. This statement should provide the following information, where applicable:

- Accession codes, unique identifiers, or web links for publicly available datasets
- A description of any restrictions on data availability
- For clinical datasets or third party data, please ensure that the statement adheres to our policy

All data that support the findings of this study are available in the manuscript, Extended Data and Supplementary Information. Raw movies and images from microscopy experiments are available from the corresponding authors upon request. The atomic coordinates used to generate the receptor model in Figure 1 are available from the Protein Data Bank under PDB ID:3UON and ID:4MQS. Source data are provided with this paper.

Research involving human participants, their data, or biological material

Policy information about studies with human participants or human data. See also policy information about sex, gender (identity/presentation), and sexual orientation and race, ethnicity and racism.

Reporting on sex and gender	N/A
Reporting on race, ethnicity, or other socially relevant groupings	NA
Population characteristics	N/A
Recruitment	N/A
Ethics oversight	N/A

Note that full information on the approval of the study protocol must also be provided in the manuscript.

Field-specific reporting

Please select the one below that is the best fit for your research. If you are not sure, read the appropriate sections before making your selection.

☒ Life sciences ☐ Behavioural & social sciences ☐ Ecological, evolutionary & environmental sciences

For a reference copy of the document with all sections, see nature.com/documents/nr-reporting-summary-flat.pdf

Life sciences study design

All studies must disclose on these points even when the disclosure is negative.

Sample size	Single-cell fluorescence imaging: For single-cell fluorescence imaging, sample size corresponded to the number of individual cells analyzed. No formal sample-size calculation was performed because the experiments involve large, independently measured cell populations where increasing the number of cells primarily reduces measurement noise rather than altering effect size estimation. Sample sizes were determined based on established practice in the field with a minimum of 3 independent experiments.<br>Other experiments (plate-reader functional assays):<br>For all functional assays performed in the plate reader, no statistical sample-size calculation was performed either. Instead, sample sizes were chosen based on prior experience with these well-established assays, where effect sizes are robust and biological variability is low under controlled culture conditions. We routinely use at least three independent biological replicates, each measured with technical replication, because these replicate numbers have historically been sufficient to detect the expected differences with appropriate statistical power and to yield consistent results across independent experiments. The chosen sample sizes therefore provide reliable estimation of mean values and variance and are standard for this experimental system.
Data exclusions	No data were excluded from the analysis.
Replication	All experiments were successfully reproduced through at least 3 independent biological replicates.
Randomization	Randomization was not relevant to this study because all experimental conditions were predefined by the study design (e.g., specific treatments such as different agonists, G proteins etc. and specific biosensor constructs) and were performed on parallel cultures of the same cell line under tightly controlled laboratory conditions. Samples were not “assigned” from a heterogeneous population but were generated directly by applying the prescribed experimental manipulation to each group. As a result, there were no covariates or sources of allocation bias that randomization would mitigate. All samples within each experiment were processed on the same day using identical protocols, which effectively controls for technical variability and ensures that group differences arise solely from the intended experimental manipulation.
Blinding	Blinding was not relevant to this study because the experimental readouts were objective, quantitative measurements (e.g., plate-reader

Blinding	signals, fluorescence imaging, or software-based analysis) that do not involve subjective assessment by the experimenter. Samples were processed in parallel using standardized protocols, and data acquisition and quantification were performed using controlled instrument settings and predefined analysis parameters. As a result, experimenter knowledge of sample identity could not influence the measurement or interpretation of the results, and blinding would not have altered the outcome of the analysis.

Reporting for specific materials, systems and methods

We require information from authors about some types of materials, experimental systems and methods used in many studies. Here, indicate whether each material, system or method listed is relevant to your study. If you are not sure if a list item applies to your research, read the appropriate section before selecting a response.

Materials & experimental systems

n/a	Involved in the study
☐	☒ Antibodies
☐	☒ Eukaryotic cell lines
☒	☐ Palaeontology and archaeology
☒	☐ Animals and other organisms
☒	☐ Clinical data
☒	☐ Dual use research of concern
☒	☐ Plants

Methods

n/a	Involved in the study
☒	☐ ChIP-seq
☒	☐ Flow cytometry
☒	☐ MRI-based neuroimaging

Antibodies

Antibodies used	An Anti-HA-Peroxidase, High Affinity from rat IgG1 monoclonal (1:1000) (Clone 3F10, Sigma-Aldrich) was used in this study. Antibody Identifier: 12013819001 RRID: AB_390917
Validation	The Antibody validation statement can be found on the website: https://www.sigmaaldrich.com/DE/en/product/roche/12013819001

Eukaryotic cell lines

Policy information about cell lines and Sex and Gender in Research

Cell line source(s)	HEK-tsA201 cells (labeled HEK293T cells in the manuscript) were purchased from Sigma-Aldrich (ECACC Cat#96121229) and HEK293AD cells were purchased from Biocat, cat. no. AD-100-GVO-CB.
Authentication	Cell lines have not been authenticated after purchase. Early passages were consistently used. Cells were undergoing verification of cell morphology during cell-culture routine.
Mycoplasma contamination	Cells were routinely tested for mycoplasma contamination using MycoAlertTM mycoplasma Detection Kit from Lonza (Basel, Switzerland). Cells have been tested negative from contamination.
Commonly misidentified lines (See ICLAC register)	No commonly misidentified cell lines were used in the study.

Plants

Seed stocks	Not relevant for this study.
Novel plant genotypes	Not relevant for this study.
Authentication	Not relevant for this study.
